# Postpartum Experiences of Early Skin-to-Skin Contact and the Traditional Separation Approach After a Very Preterm Birth: A Qualitative Study Among Mothers

**DOI:** 10.1177/23333936221097116

**Published:** 2022-05-19

**Authors:** Anne Marit Føreland, Helene Engesland, Laila Kristoffersen, Liv Fegran

**Affiliations:** 1 Hospital of Southern Norway, Kristiansand, Norway; 2 University of Agder, Kristiansand, Norway; 3 St. Olav’s University Hospital, Trondheim, Norway; 4 University of Science and Technology, (NTNU), Trondheim, Norway

**Keywords:** very preterm birth, mothers’ experience postpartum, early skin-to-skin contact, bonding, qualitative research, Norway

## Abstract

Traditional care immediately after very preterm birth separates the mother and child by the transfer of the infant to the neonatal intensive care unit. A nonseparation approach is currently being considered, allowing early skin-to-skin contact in the delivery room/postoperative care unit. This study aimed to explore mothers’ experiences of early skin-to-skin contact and traditional care. A qualitative study using individual semi-structured interviews with five mothers from each of the two groups was conducted. Content analysis revealed that both groups’ experiences were characterized by (i) mothers’ need to be affirmed of their infants’ vitality, (ii) bonding challenges, and (iii) benefits of skin-to-skin contact. We suggest that early skin-to-skin contact after very preterm births is crucial for the bonding process and mothers’ feelings of safety and well-being. When early skin-to-skin contact is infeasible, our findings reveal the significance of photos, information, and the father’s presence at the time of postpartum separation.

## Background

Over the past four decades, there has been a change in the way infants are treated after birth. Instead of being dressed and put directly into a cot, healthy full-term newborns are now being placed in a skin-to-skin position with their mothers immediately after birth. Skin-to-skin contact (SSC) was first implemented after vaginal birth, but studies have also recommended this procedure after cesarean section (Hubbard & Gattman, 2017; Schneider et al., 2017; Stevens et al., 2014). The intervention is defined as placing the infant, only wearing a diaper, in an upright position on the bare chest of their parent (Campbell-Yeo et al., 2015). SSC is also recommended for premature infants (born before 37 completed weeks of gestation (World Health Organization [WHO, 2018]), even if they are in need of medical care in a neonatal intensive care unit (NICU) (WHO, 2003). This kind of care is also called kangaroo mother care (KMC) and originates from Bogotá in Colombia (Whitelaw & Sleath, 1985), where the premature infant was placed on their mother’s chest because of a lack of equipment and staff. The method produced several unforeseen benefits, such as a more stable body temperature, earlier breastfeeding, and improved bonding between the mother and the infant (Whitelaw & Sleath, 1985). These findings have been confirmed in several studies, and SSC has become a common practice in the NICU worldwide due to the physiological and psychological benefits to the health of both the infant and the mother (Campbell-Yeo et al., 2015; Jones & Santamaria, 2018; Stevens et al., 2014). SSC is performed for a shorter or longer period—often referred to as intermittent, continuous, immediate, or early SSC, depending on the time of initiation and duration (Campbell-Yeo et al., 2015; WHO, 2021).

When the infant is born very preterm (gestational age 28–32 weeks; WHO, 2018), the traditional procedure is to separate the mother and the infant immediately. While very preterm infants are transferred to the NICU immediately following birth, mothers remain in the delivery room or postoperative care unit. Low gestational age is perceived as one of the barriers to initiating early SSC (Niela-Vilén et al., 2013). Other barriers include safety concerns, a lack of staff and guidelines, established routines, a lack of knowledge, and health professionals’ reluctance to change (Hubbard & Gattman, 2017; Maniago et al., 2020; WHO, 2003). However, in recent years, several studies have confirmed the safety, feasibility, and benefits of early SSC for moderately preterm infants from 32 weeks of gestation (Kristoffersen, Stoen et al., 2016). Recently, quantitative studies have explored the safety and efficacy of early SSC for premature infants born even earlier (Kristoffersen, Støen et al., 2016; Linnér et al., 2020; Mehler et al., 2020; WHO, 2021).

Through his attachment theory, Bowlby (1969) was the first to assert that children instinctively seek proximity and contact. He described the first 6 months of life as being crucial for the bond between the mother and the child. Later, psychologists Klaus and Kennell (1976) elaborated on Bowlby’s theory and hypothesized that the infant’s first hours after birth are crucial for the establishment of the bond between the mother and the infant. Other studies have emphasized the importance of this early contact and have named this time “the sacred hours” or a “sensitive period” (Dahlø et al., 2018; Klaus et al., 1972; Mehler et al., 2011). Lately, Widström et al. (2019) concluded that routines should protect this early contact as much as possible. Contact in this sensitive period has been shown to be especially significant for the attachment process in very preterm births because the mother and the infant are both in an abnormally stressful situation (Mehler et al., 2011).

So far, only one study has described the effect of early SSC versus brief visual contact between very preterm infants and mothers in the delivery room (Mehler et al., 2020). That randomized controlled trial found a decreased risk of bonding problems and maternal depression at 6 months in the group who had early SSC in the delivery room. To our knowledge, a qualitative study exploring mothers’ experience from early SSC after a very preterm birth is lacking. Therefore, the aim of the present study was to explore mothers’ experiences of two different approaches within the first hours after a very preterm birth: (i) staying together with the infant, in a skin-to-skin position, in the delivery room/postoperative care unit for 2 hours after delivery (“early SSC”) and (ii) traditional care (“TC”), where the infant is transferred to the NICU immediately following delivery while the mother remains in the delivery room/postoperative care unit.

## Method

### Design

A qualitative design was used to explore mothers’ experiences after a very preterm birth. Inspired by a phenomenological-hermeneutic approach, semi-structured interviews were used to obtain in-depth knowledge of the mother’s experiences (Brinkmann & Kvale, 2015). This qualitative study was conducted as an elaboration of a multicenter randomized controlled trial including three hospitals in Norway (Kristoffersen, Støen et al., 2016). The qualitative part was intended to establish a more comprehensive picture from the participating mothers’ point of view, a perspective not covered by the quantitative part of the study (Polit & Beck, 2020)

### The Randomized Controlled Trial: Early Skin-to-Skin Contact or Incubator for Very Preterm Infants

The present study invited mothers who had already participated in the ongoing randomized controlled trial (Kristoffersen, Støen et al., 2016). The aim of the randomized controlled trial was to explore the safety and feasibility of early SSC after a very preterm birth and to measure the subsequent effect on cognitive scores, infant physiological stability, and maternal mental health. The inclusion criteria were Norwegian-speaking mothers giving birth at gestational age 28 to 32 weeks vaginally or via cesarean section. The exclusion criteria were infant birth weight <1,000 g, infants in need of intubation or oxygen >40% at 20 minutes of age, and severe congenital malformations. Mothers under general anesthesia were excluded. After initial stabilization, infants were randomized to the intervention receiving early SSC in the delivery room/postoperative care unit or to TC, being transferred to the NICU and separated from their mothers (Kristoffersen, Støen et al., 2016). The inclusion and randomization were done by a head consultant and a neonatal nurse. There were no restrictions in the TC group to perform SSC when mothers came to the NICU from the delivery room/postoperative care unit.

### Recruitment, Setting, and Participants

When obtaining consent for the randomized controlled trial before delivery, the mothers were informed that they would be invited to participate in the qualitative study after delivery. The present study was conducted at one of the three hospitals participating in the randomized controlled trial. This was a regional hospital with a 13-bed NICU at Level 3a, treating preterm infants from 28 weeks of gestation (Helsedirektoratet, 2019). All mothers included in the randomized controlled trial at this hospital were consecutively invited to participate in the qualitative study. They were approached 1 to 4 weeks postbirth, when the mother and the infant were in a stable situation. Nine out of ten participants were recruited from the same hospital. However, due to a small number of very preterm deliveries and a subsequent slow recruitment, one mother was recruited from a second hospital affiliated with the same study. This was a university hospital with an NICU at Levels 3 a, b, and c, treating the smallest and most fragile newborns (Helsedirektoratet, 2019).

### Data Collection

The interviews were carried out between May 2017 and March 2020, 2 to 4 weeks after delivery. The interview process was inspired by Brinkmann and Kvale (2015) in terms of preparation of the questions, assessment of the performance, and the transcription of the interviews. The face-to-face interviews took place in a family room in the NICU without disturbances and were conducted by one of three nurses in the study group, mainly the first and second authors. The interviews began with an opening question allowing the participants to talk freely about their experiences of the phenomenon being explored, as described by Brinkmann and Kvale (2015). This initial question in the interview guide was as follows: “Can you tell us about your experience of the first hours after the infant was born?” To elaborate or clarify specific experiences, we used follow-up questions addressing issues related to the first time the infant was placed on the mother’s chest, their experience of the time before this event, and their thoughts of the father’s experience. The interviews lasted between 20 and 44 minutes (mean = 33 minutes), and all interviews were audio-recorded and transcribed verbatim.

### Ethical Considerations

Ethical approval was obtained from the Regional Ethical Committee (no. 2013/638/REK midt). The study protocol was approved by the research department at the hospital. Informed consent was obtained twice: (i) for the randomized controlled trial before delivery and (ii) for the qualitative study 1 to 4 weeks after delivery, in the NICU. Both times, the mothers received verbal and written information about the study, and we assured them that there was enough time to make a considered decision before consenting.

Data were stored in accordance with guidelines for storing research material, and the audio recordings were deleted after transcription (Brinkmann & Kvale, 2015). The researchers worked as nurses in the NICU during the study period and avoided close contact with the participating mothers before the interviews. Some of the mothers were severely emotionally affected during the interviews. In this situation, we paused and gave the participants the opportunity to finish speaking. The ethical issue of continuing these interviews was also discussed within the research team. To take care of the participants, we decided to prepare colleagues in the NICU to be available for the participants after the interviews (Brinkmann & Kvale, 2015).

### Data Analysis

Qualitative content analysis, as described by Graneheim and Lundman (2004), was performed. The transcribed interviews were imported into the data management software program NVivo 11 (QSR International, Melbourne, Au.). Data from both groups of participants were analyzed together, but separation of data into early SSC and TC subcategories visualized the differences and similarities of the two groups. First, all the transcripts were read entirely by the first and second authors several times to obtain a sense of the whole. Next, the transcripts were structured into condensed meaning units consisting of text units describing manifest content related to the same central meaning (Graneheim & Lundman, 2004). The abstraction and sorting of meaning units into tentative categories were discussed frequently between the coauthors until consensus was reached. Finally, after moving back and forth several times, the categories were abstracted into three themes to formulate the latent meaning of the text (Graneheim & Lundman, 2004).

## Results

Of the 15 mothers eligible for inclusion, two denied participation, one was transferred to another hospital, and two were excluded due to a lack of language skill qualifications. The sample consisted of five mothers from each group. Eight out of ten included mothers were ethnic Norwegians, but mothers from three different continents and with various educational backgrounds were represented. The delivery method was cesarean section (seven mothers) and vaginal delivery (three mothers). Some mothers experienced an emergency delivery, while others were prepared for very preterm delivery as inpatients for several days or weeks. The infants’ mean gestational age was 30 weeks and 4 days, and their mean birth weight was 1,486 g. Five participants were first-time mothers, while the other five had previous experience with preterm birth, stillbirth, or adoption. Characteristics of the sample are presented in Table 1.

**Table 1.** Characteristics of the Sample.

Mothers *n* = 10	
Age range	24–38 years (mean = 33.4)
Education	
High school	*n* = 5
College/university	*n* = 4
Not available	*n* = 1
Ethnicity	
Asian	*n* = 1
South American	*n* = 1
European (Norwegian)	*n* = 8
Previous births	4
Previous preterm births	3
Previous stillbirth	1
Adopted previous child	1
This birth	
Cesarean	7
Vaginal	3
Infants (*n* = 10)	
Gestation period range (weeks)	29 + 2 – 31 + 4 (mean = 30 + 4)
Sex	
Female	4
Male	6
Birth weight (g)	1,100–1,915 (mean = 1,486)
Type of care received	
Early skin-to-skin contact [early SSC]	5
Traditional care [TC]	5
Hours after birth when first skin-to-skin contact	
Early skin-to-skin contact group	1 (mean = 1)
Traditional care group	2–30 (mean = 8.8)

Through data analyses we identified three themes: (i) mothers’ need to be affirmed of their infants’ vitality, (ii) mother–infant bonding challenges, and (iii) the benefits of SSC.

### Mothers’ Need to be Affirmed of Their Infants’ Vitality

Giving birth to a very preterm infant created a high level of stress and anxiety, often described as a dramatic experience. In this challenging situation, the main concern of all mothers was the condition of the infant. Mothers in both groups were worried whether the infant was alive and healthy. The first sign of a living child was a strong emotional experience, often expressed during the interviews with tears. The very first cry, sight, and touch were affirmations of life and were described as “magic” by a mother: “When I heard her first little cry, I was so happy that she was alive!” (M [early SSC]).

After delivery, the mothers were transferred to the postoperative care unit or remained in the delivery room. While infants of mothers in the early SSC group were placed on the mother’s chest, mothers in the TC group were separated from their infant for the following 2 to 30 hours, depending on the mother’s medical status and the need for observations following delivery. Some mothers in the TC group did not even get a glimpse of their infant before the newborn was transferred to the NICU. During the time of separation, mothers worried and wondered about the stability and appearance of their infant: “Is she all right? What are they doing to her?” (M [TC]). All informants in the TC group described how information about the infant’s vitality reassured them. The worst situation was when they did not know what was happening. The mothers were comforted by photos of the infant, especially printed photos. However, they were still longing for physical contact with their infant, as one mother expressed, “A photo is helping. However, the best is to hold your living child!” (M [TC]).

Most mothers in the TC group described this separation period as a time filled with frustration, fear, and sometimes guiltiness. Some mothers experienced a feeling of hours passing slowly, while others perceived time flying or the time aspect was unclear. Some of the mothers felt tired and exhausted; one of them was critically ill. All mothers in the TC group mentioned that they tried to keep a positive thinking approach during the separation, as described by one mother: “I knew he was in safe hands. I made it up as it went along” (M [TC]). The well-being and safety of the infant was uppermost in the mothers’ minds, and they were confident that the physicians and the nurses chose the best treatment for them and their infant. Looking forward to the reunion gave comfort and helped them to endure the hours of waiting. One mother [TC] stated that the first SSC took place not too long after delivery, even though she was not in the early SSC group. Another participant [TC] expressed that TC might be best in the case of infant instability. If the infant had been placed in a skin-to-skin position immediately after delivery, she was afraid that the infant could be physically unstable and would have to be taken away from her. She explained that disrupted contact might provoke fear and subsequent bonding challenges.

None of the mothers in the intervention group described feelings such as fear, insecurity, or worries about their infant’s medical condition during early SSC. After initial stabilization of the infant following delivery, mothers in the early SSC group had their infant in a skin-to-skin position within 1 hour. They highly appreciated staying together, as early SSC was the best way to affirm that the infant was alive and all right. They were so close that they could experience the vitality of the infant by their own senses, as one mother expressed with tears constantly running down her cheeks: “I can feel her breathing. She is alive! Early skin to skin is the best that ever happened to me” (M [early SSC]).

In addition to the affirmation of the infant’s vitality, all mothers in the early SSC group described how adequate information and the constant presence of nurses contributed to the feeling of safety. Moreover, the presence of physicians and midwives when needed during this period was appreciated. The mothers talked about the staff’s competence in performing the early SSC procedure in combination with the large amount of medical emergency equipment. One mother noted, “The purpose of the different equipment was explained to me. I knew he was well monitored and taken care of, so I was not afraid” (M [early SSC]).

### Mother–Infant Bonding Challenges

Mother–infant bonding was experienced as challenging in both groups. The informants described feelings of becoming a mother with words such as surreal, unreal, strange, and shocking. Early SSC promoted bonding, as described by one mother comparing this birth with a previous preterm birth: “I think I got the motherhood feelings much earlier this time because of early SSC” (M [eSSC]).

Infants in the TC group were separated from their mothers and transferred to the NICU after delivery. Several of the informants perceived the infant in the incubator as lonely, tiny, naked, ill, or covered with technical equipment. This brought out a sense of strangeness and fright, an unfamiliar state characterized as “a cold feeling” and “a feeling of separation.” However, some of the infants were placed on the father’s chest in the NICU when the TC mothers were still in the postoperative care unit. This was perceived as comforting for the mother, and one mother emphasized the importance of the father’s presence concerning father–infant bonding: “I am convinced that the bonding between the father and the infant will be enhanced” (M [TC]). The same mother also appreciated the exchange of a cloth with the smell of the infant or mother and the possibility of providing expressed milk: “It was a feeling of being a mom, even though I am here and not with him!” (M [TC]).

Mothers in both groups valued the presence of the father. Fathers in the TC group were described as messengers contributing to comfort and company, going back and forth between the infant and the mother. In the early SSC group, the informants appreciated being united as a family and being able to explore and sense the baby together as newly fledged parents: “Maybe both the mother and the father get the feeling that this is *our* baby, not just an infant inside an incubator” (M [early SSC]).

### The Benefits of Skin-to-Skin Contact

Mothers in both groups described the first time of SSC as a positive experience, even if this event occurred early for the early SSC group or later, after several hours of separation, for the TC group. It was an overwhelming emotional experience. The first SSC was frequently pointed out as *the* highlight of the hours around labor. It was compared with “the first love.”

Mothers in both groups pointed out that the first SSC had a positive effect, both physically and emotionally. They felt calmer and gave various rich descriptions of physical improvements, such as reduced blood pressure, less nausea and shivering, normalizing blood tests, lactation stimulation, less pain, reduced stress, and less exhaustion. Their worries were replaced with feelings of happiness and well-being, as one mother expressed, “When they passed him over to me, I forgot everything else” (M [early SSC]). She described a feeling of her body being filled with a healing warmness.

The mothers perceived that the infant appeared to be stable and calm during the first SSC session. They described that the infant was physiologically stable, the breathing pattern was stable, and the infant’s need for oxygen decreased. One mother said, “She settled down and was relaxed; it seemed to be a pleasure for her!” (M [early SSC]). Some mothers thought that being in a skin-to-skin position was soothing and was something familiar to the infant because they recognized the voice, smell, and heartbeat of the mother.

## Discussion

This study provides insight into mothers’ experiences of the nonseparation approach early SSC following very preterm delivery and of the traditional approach, in which infants are separated from their mothers. These findings shed light on the new intervention and TC, revealing mothers’ need to be affirmed of their infants’ vitality, bonding challenges, and benefits of SSC.

### The Significance of Affirmation of Vitality

All mothers were concerned about the vitality of the infant. Our findings agree with other studies describing mothers’ fear of preterm survival and health (Baum et al., 2012; Mu et al., 2020). The reason affirmation of vitality was so essential and was expressed with tears might be connected to the life-threatening situation of the mother and the infant. Vaerland et al. (2018) described how mothers giving birth at a very early stage of pregnancy reflected that they and their infant could have died. A stillbirth experience and bad memories from previous preterm deliveries made this moment even more dramatic for some of the mothers in our study. The first contact within the very first hours has been shown to be important, especially after very preterm births (Mehler et al., 2011, 2020). Early SSC could be a measure for reducing maternal anxiety—where mothers experience, with their own senses, that the infant is doing well (Mehler et al., 2020). However, if the mothers in our study were separated from the infant, information and photos of the infant contributed to their consolation. Nevertheless, the mothers who had early SSC recognized this approach as the optimal way of being affirmed of the infant’s well-being and vitality.

Mothers in both groups recalled the situation as frightening and unfamiliar. Mothers in the TC group who were separated from their infants tried to cope with the challenges by using positive-thinking strategies. Our findings are supported by research describing how mothers rely on physicians and nurses’ knowledge of what is best for the infant (Spinelli et al., 2016). One mother in the TC group considered that TC might be a safer intervention than early SSC due to the risk of unexpected infant instability during the early SSC session. Safety concerns have been shown in other studies as obstacles to early SSC (Hubbard & Gattman, 2017; Maniago et al., 2020). However, all mothers who received early SSC in our study described a feeling of safety by mentioning crucial aspects such as adequate information, monitoring, and the constant presence of health professionals who followed a detailed early SSC procedure and were close by the whole time. A recent review by Maniago et al. (2020) underlines many of the same aspects to improve utilization of SSC (named as Kangaroo Mother Care): “adequate manpower, clear guidelines, sufficient supplies and equipment, capacity buildings among staff and proper kangaroo mother care information dissemination for patients” (p. 293). One question in our interview guide referred to whether the mothers in the early SSC group worried about having their very preterm infant in a skin-to-skin position when the infant was connected to several medical-technical equipment. None of them were worried or afraid. They argued that this treatment was the best for the infant. This might be influenced by their former experience with such medical equipment. However, Dahlø et al. (2018) indicated how mothers’ feeling of safety is supported by calm nurses taking care of medical equipment and assessing the infant’s condition. Maniago et al. (2020) highlighted how nurses may receive education and support on how to safely assist parents in this situation. This is essential when facilitating early SSC and indicates an important implication for future practice.

### Bonding After a Very Preterm Birth

Mothers in both groups described challenges regarding bonding when one becomes a parent after a very preterm birth. The mothers in the TC group demonstrated strategies to promote bonding even in the time of separation. They kept the long-distance contact with the infant through photos, information, or a cloth with the smell of the infant or mother, or by expressing milk. These strategies could constitute some form of resilience in the face of an adverse situation, where the mother is separated from their newborn. Similar strategies have been reported by other studies (Lindberg & Öhrling, 2008; Værland et al., 2017). Conversely, for mothers in the early SSC group, having the infant close by helped with bonding.

Vague motherhood feelings were reported in our study. This aspect has been explained by mothers’ truncated pregnancy, traumatic delivery, or immediate separation after the delivery (Lindberg & Öhrling, 2008; Maastrup et al., 2018; Vaerland et al., 2018). “No longer pregnant, not yet a mother” is a descriptive title of one article (Baum et al., 2012). An important aspect is the lack of the 2 to 3 months of pregnancy that is crucial for psychological maternity formation (Spinelli et al., 2016; Stern et al., 1998). The attachment process is also influenced by the reciprocal response from the infant (Fegran et al., 2008). Bowlby (1969) described how the infant instinctively searches for contact. This response might be difficult to detect in an immature, vague response from very preterm infants (Als, 1982). Proximity through touch and visual contact is shown to be important in this fragile process to develop bonds between the parent and the infant (Fegran et al., 2008; Klaus & Kennell, 1982; Maastrup et al., 2018). The very first hours are hypothesized to be of special value, characterized as sacred hours or a sensitive period, and physicians and nurses should protect this time as much as possible (Dahlø et al., 2018; Mehler et al., 2011). Contact in the first 3 hours has been shown to help form an important basis for secure attachment of the preterm infant (Mehler et al., 2011, 2020).

Mothers in the TC group highly appreciated the presence and the role of the father, who was a connection between the mother and the infant. Some mothers in the TC group were comforted by knowing that the infant had SSC with the father in the NICU, an experience also promoting fatherhood feelings and bonding. This is also shown in previous research (Maastrup et al., 2018; Vaerland et al., 2018). However, the fathers struggled with the choice of staying with the infant or the mother. Værland et al. (2017) also described this challenge. Conversely, mothers in the early SSC group expressed gratitude for the continuous presence of the father during the 2 hours of early SSC. They described a feeling of being undisturbed as a family, despite many professionals and all the medical equipment in the room. Early SSC makes the bonding process possible as a common family experience from the very beginning, even after a very preterm birth. This approach is in accordance with current recommendations that facilitate time together for the family and minimize separation in situations where the mother and the infant both need intensive care (Gardner & Voos, 2020).

### Positive SSC Experiences: How Urgent Is the Initiation?

Mothers in both groups gave overwhelming positive descriptions from the very first SSC, independent of the time of waiting. They described SSC as an approach that calmed and stabilized them and their infant, both physically and emotionally. Our findings are in accordance with other studies describing the benefits of SSC (Campbell-Yeo et al., 2015; Jones & Santamaria, 2018; Stevens et al., 2014). How urgent is the initiation of SSC? Does it matter whether SSC starts within the first hour (early SSC) or later by the first meeting in the NICU (TC)? The first hour of life, named “the golden hour” by Reynolds et al. (2009), plays a critical role in the outcomes of low-birth-weight infants. Reynolds et al. (2009) described how neonatologists and nurses optimized physical stabilization at this early stage with a focus on preventing complications. Early SSC might be considered beneficial as part of this initial treatment. If the mother is stabilized and relieved as well, as experienced by mothers in our study, it is even more significant. Physiological aspects of early SSC for the mother and the infant after very preterm birth is a current issue. Recent and ongoing quantitative studies are extending knowledge in this aspect (Kristoffersen, Støen et al., 2016; Linnér et al., 2020; Mehler et al., 2020; WHO, 2021).

One mother in our study articulated that the time of separation was not long, indicating that the TC procedure was not vastly different from early SSC. Three mothers in our study waited for 4 hours—which is quite a usual separation time after very preterm deliveries, according to our experience. However, these mothers did not gain the previously described benefits from the important initial sacred hours. Another aspect is that the separation time could be extended. One mother in our study did not see or touch her newborn for 30 hours after the birth. Our findings are in accordance with other studies in which unexpected events or critical health conditions related to the mother or the infant resulted in a long separation time (Maniago et al., 2020; Vaerland et al., 2018). Maastrup et al. (2018) showed the value of experiencing SSC in a case where extremely preterm twins did not survive, indicating “it is important to avoid delaying the initiation of SSC” (p. 551). Stadd et al. (2020) also highlighted this aspect. Two informants in our study described the significance of SSC before unexpected, critical situations occurred.

Implementing early SSC following very preterm delivery in practice demands dedication and effort, and nurses are considered to have a key role in the implementation of practices and policies (Maniago et al., 2020). Of all the implementation obstacles Maniago et al. (2020) mentioned, we highlight nurses’ reluctance as a core factor. Early SSC challenges the NICU routines, and a creative problem-solving attitude is needed to establish the new approach (Boyd, 2017). From recent research publications and our own experiences as NICU nurses, we have seen early SSC realized in situations we thought were impossible (Mehler et al., 2020; WHO, 2021).

Even when early SSC within the first hour postpartum is infeasible, it might be possible in TC to reduce the mother–infant separation time. Improved collaboration between nurses, midwifes, pediatricians, and anesthetists is recommended to reduce the separation time (Hubbard & Gattman, 2017; Niela-Vilén et al., 2013).

## Methodical Considerations and Limitations

A qualitative design was chosen because we wanted to elaborate on the findings from the affiliated randomized controlled trial by gaining insight into the mothers’ experiences. Generalizing from a qualitative study is a limitation due to the small number of informants. The strength of our study is that it gives in-depth knowledge from an intervention that is little explored after very preterm birth, as well as from the most common routines (TC). Findings from both groups might be transferable and interesting for nursing practice.

The cultural backgrounds of the included mothers might have influenced the results. There might have been misunderstandings in two of the interviews due to not using their native language. A lack of language qualifications was the reason two mothers were excluded; however, multiple cultural backgrounds might have given a broader insight into the phenomena from an international point of view. Mothers’ different experiences from former deliveries, their education, and preparations for vaginal or cesarean delivery might have influenced the results. However, the different backgrounds were spread quite equally within the two groups and may conduce to diversity of the sample.

Being interviewers and clinical nurses in the same NICU implied some challenges. We did not have close contact with the included mothers before the interviews to avoid promoting a feeling of “pay back” in the mothers. Our preconception was influenced by our affiliation to the ward, experience as NICU nurses, and enthusiasm for the new intervention. We maintained consciousness of this challenge by reminding each other regularly to be open to all aspects through the process of planning, interviewing, and analyzing. The personal knowledge of the ward was an advantage because it made it easier for us to arrange the interviews, find relevant questions, and handle mothers’ emotional reactions.

We made reflective notes on the research process to support the analyses and the writing of the paper. This is a way to provide trustworthiness (Graneheim et al., 2017). Several discussions and reflections were held between the authors throughout the whole process. We obtained comprehensive data for our analysis, and we have attempted to thoroughly describe every step of the study. Other researchers might have made other appraisals and interpretations (Graneheim & Lundman, 2004). Other informants may have given data different from ours. Nevertheless, we consider our process proper and our findings relevant for future nursing practice.

## Conclusions and Implications for Practice

Our findings of mothers’ experiences show their strong need for affirmation of vitality immediately after a very preterm delivery. Affirmation was best obtained through SSC. Early initiation, as shown by early SSC, promotes the bonding process and mothers’ feeling of safety and well-being. Considering our findings in the light of bonding theories about the sensitive period and the fragile bonding process following very preterm delivery, we suggest early SSC to be even more crucial and rewarding compared to deliveries later in pregnancy. When early SSC is infeasible, our study shows the significance of minimizing the separation time and demonstrates the value of photos, information, and the father’s presence in the first postpartum hours. These latter findings from the TC group and findings from the early SSC group contribute to important implications for nursing practice to provide the optimal care of mothers after very preterm deliveries.

The high technology and security circumstances in the Norwegian context can be seen as unobtainable in low-resource settings. Nevertheless, early SSC can be considered a relevant approach in various settings, given the positive results from the original study conducted in Bogotá (Whitelaw & Sleath, 1985) and recent research from low-resource hospitals (WHO, 2021). When there is a lack of technical equipment, such as incubators, these studies have highlighted that mothers might give the same and even better treatment than incubators by laying the newborn naked on their chest. In our experience, it is essential to explore possibilities of promoting early mother–infant contact, as well as perform careful and exact planning and implementation of new approaches. More research is needed to investigate the feasibility and safety of early SSC after very preterm births in different settings.
